# QoS-based management of biomedical wireless sensor networks for patient monitoring

**DOI:** 10.1186/2193-1801-3-239

**Published:** 2014-05-09

**Authors:** Carlos Abreu, Francisco Miranda, Manuel Ricardo, Paulo Mateus Mendes

**Affiliations:** Instituto Politécnico de Viana do Castelo, Viana do Castelo, Portugal; CIDMA, Universidade de Aveiro, Aveiro, Portugal; INESC TEC, Faculdade de Engenharia, Universidade do Porto, Porto, Portugal; Centro Algoritmi, Universidade do Minho, Braga, Portugal

**Keywords:** Patient monitoring, Quality of service, Admission control, Biomedical wireless sensor networks

## Abstract

Biomedical wireless sensor networks are a key technology to support the development of new applications and services targeting patient monitoring, in particular, regarding data collection for medical diagnosis and continuous health assessment. However, due to the critical nature of medical applications, such networks have to satisfy demanding quality of service requirements, while guaranteeing high levels of confidence and reliability. Such goals are influenced by several factors, where the network topology, the limited throughput, and the characteristics and dynamics of the surrounding environment are of major importance. Harsh environments, as hospital facilities, can compromise the radio frequency communications and, consequently, the network’s ability to provide the quality of service required by medical applications. Furthermore, the impact of such environments on the network’s performance is hard to manage due to its random and unpredictable nature. Consequently, network planning and management, in general or step-down hospital units, is a very hard task. In such context, this work presents a quality of service based management tool to help healthcare professionals supervising the network’s performance and to assist them managing the admission of new sensor nodes (i.e., patients to be monitored) to the biomedical wireless sensor network. The proposed solution proves to be a valuable tool both, to detect and classify potential harmful variations in the quality of service provided by the network, avoiding its degradation to levels where the biomedical signs would be useless; and to manage the admission of new patients to the network.

## Introduction

Patients’ monitoring in general or step-down hospital units is crucial to avoid clinical worsening of inpatients ([Alemdar and Ersoy 2010]; [Nouira and Trabelsi 2012]). However, in most cases, despite the necessity of continuous monitoring, nursing professionals measure vital or physiological signs (e.g., temperature, pulse and respiratory rates, blood pressure or oximetry) manually a few times a day. Such episodic measurements can be complemented by efficient and reliable real-time monitoring systems, bringing out an enhancement of the quality of care, while freeing the nursing staff to provide extra attention to the inpatients ([Chipara et al. 2010]; [Ko et al. 2010b]).

Depending on its application and purpose, real-time patient monitoring systems have distinct requirements ([Baig and Gholamhosseini 2013]). In one hand, continuous monitoring of cardiac or cerebral signs (i.e., electrocardiogram or electroencephalogram, respectively) requires high throughput networks (e.g., IEEE 802.11-based networks). On the other hand, gathering of vital or physiological signs can be done through Biomedical Wireless Sensor Networks (BWSNs) based on the IEEE 802.15.4 standard ([Ko et al. 2010a]). BWSNs are distinct from IEEE 802.11-based wireless networks in several aspects. Unlike IEEE 802.11 networks, they form a distributed, self-organised and energy-efficient low data rate network without the need of a physical infrastructure. Due to its flexibility and low energy consumption, BWSNs have been successfully used in several patient monitoring scenarios ([Alemdar and Ersoy 2010]; [Honeine et al. 2011]).

**Figure 1:**
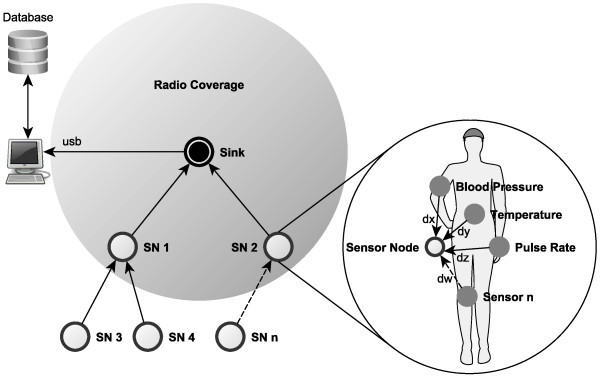
**Biomedical Wireless Sensor Network for patient monitoring in hospital environments.**

The main goal of a BWSN is to collect recorded data and send it to a central database to be integrated in the hospital information system and, then, used by the healthcare professionals. In this way, due to the critical nature of the carried data, BWSNs have to fulfil appropriate Quality of Service (QoS) requirements, which depend on both, its application and its purpose ([Baker and Hoglund 2008]). More, regarding realistic deployments, BWSNs have to transport distinct data types while providing the required QoS to all of them. Figure 1 represents a patient monitoring system, where each sensor node can generate several data flows, each one with its specific QoS requirements (depending on the intrinsic characteristics of the corresponding vital or physiological signal, or on the patient’s health condition).

The QoS requirements of BWSNs are, typically, defined at the earliest stages of the project and addressed across the different layers of the communication stack. However, due to the dynamic nature of hospital environments BWSNs can be exposed to hostile situations regarding the radio communications. Consequently, the QoS provided by the network can change very often in an unpredictable way ([Ko et al. 2010b]). In such harsh environments the QoS degradation can be related with either random or deterministic factors. Random factors, such as the dynamics of the network or hospital environment, the radio interferences, or the patients’ mobility need to be monitored and classified in order to detect situations in which they can cause a severe degradation of the QoS required by the applications using the BWSN. Deterministic factors, such as the network congestion due to the overpopulated network can be avoided by using QoS assessment and admission control tools to deploy the nodes in appropriate areas. In this context, this paper contributes with a QoS-based management system that can be used both, to assess the QoS provided by the BWSN and to assist healthcare professionals managing the admission of new sensor nodes (i.e., new patients to be monitored) to BWSNs while knowing its impact on the QoS provided by the network.

In what follows, some related works are outlined in Section “Related work”, and then the proposed QoS-based network management system is presented in Section “Proposed QoS-based network management system”. Section “Time domain analysis of the QoS metrics” presents the methodology used to detect and classify QoS degradation events. In Section “Experiments and results”, the proposed QoS-based network management system is assessed and the results analysed. Finally some conclusions are drawn.

## Related work

The emergence of WSNs as an important communication infrastructure regarding the development of real-time monitoring applications, such as patient monitoring, increases the demand for both QoS monitoring systems and admission control systems. As discussed in ([Pereira et al. 2012]) and ([Lindh and Orhan 2009]), both QoS monitoring systems and admission control systems are keystones to achieve high levels of reliability and performance in WSNs; in fact, they complement each other.

In the last few years, a few approaches have been proposed for both QoS monitoring and admission control for WSNs. The authors of ([Sun et al. 2005]) propose SenProbe, an end-to-end capacity estimation tool that can be used to performance analysis and network deployment planning. SenProbe uses one-way active measurements to estimate the end-to-end path capacity by injecting packet trains in the network to measure the packet dispersion. As an alternative to real-time active measurements, the authors of ([Yang and Kravets 2005]) propose a contention-aware admission control protocol designed for IEEE 802.11 ad-hoc networks, which estimates the available channel capacity by allowing each node to measure the time that the communication channel is busy. The work presented in ([Orhan and Lindh 2011]) offers a measurement-based performance and admission control system, where the admission decision is made by the network coordinator, based on real-time measurements of the packet loss ratio provided by a performance meter. The performance meter runs on each sensor node and it is continually tracking the number of sent and received data packets and bytes. By running on each sensor node, the performance meter contributes to its energy depletion and consequently reduces the network lifetime. Since WSNs are composed, typically, of energy-constrained sensor nodes, the increase of energy consumption on their nodes must be avoided. In fact, depending on the network application it could be a strong weakness. Although proposing different solutions, all these studies support the importance and need of using both QoS monitoring and admission control systems, to manage and improve the performance of WSNs.

This paper proposes a new QoS-based network management system for BWSNs comprised by two modules, namely the QoS monitoring module and the admission control module. The QoS monitoring module collects relevant information about the network’s performance and uses it to detect and classify QoS degradation events. Based on this information, reports are generated to inform the healthcare professionals, or who is in-charge, about the network’s performance. On its turn, the admission control module uses a probe-based admission control procedure to decide where a new sensor node can join the network. The decision where to admit the new sensor node on the network is based on time-domain analysis of the performance metrics used to assess the QoS provided by the network, namely the packet reception ratio. Within the proposed method, the probe-based procedure uses a “virtual sensor node” called QoS Probe to mimic the presence of a new real sensor node on its neighbourhood. The use of a virtual sensor node” enables to assess the network performance from a remote location, in order to decide about the best location to admit the new sensor node within the network. To avoid the problem of energy depletion in the energy-constrained sensor nodes, the proposed QoS-based network management system collects all the data required for decision making at the sink that, generally, is connected to the power line. Regarding the energy consumption in the sensor nodes, the QoS Probe is required to run only on request and for short time periods, being the extra energy consumption insignificant.

## Proposed QoS-based network management system

Regarding the BWSNs used within the suggested QoS-based network management system, this section presents the software used by its nodes. Namely, its architecture and components are described.

### Software architecture

**Figure 2:**
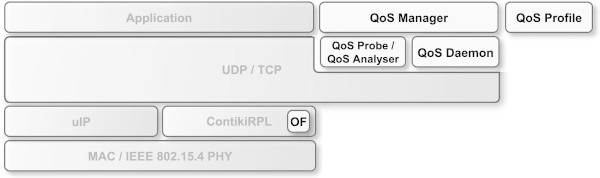
**Architecture of the software running on the BWSN’s nodes.**

The software developed to implement the proposed QoS-based network management system was built on top of the Contiki OS ([Dunkels et al. 2004]). The software running on the BWSN’s nodes follows the architecture presented in Figure 2. The QoS-based network management system adds the following modules to the base operating system architecture: QoS Manager, QoS Profile, QoS Probe, QoS Analyser and QoS Daemon. The QoS Manager is the interface between the application and the QoS-based network management system. It is responsible to manage the interactions between all the remaining components. The application uses the QoS Manager to receive information about the QoS provided by the network or to trigger actions, such as the admission control procedure. The QoS Profile contains all the relevant information about each data flow including the required QoS. The QoS Probe (i.e., the “ virtual sensor node”) runs only on the sensor nodes. It can be configured, on demand, to perform remote tasks. Such tasks include generating specific data flows or collecting information about the node (e.g., its radio link quality). The QoS Analyser runs only in the sink node and extracts the relevant information about each data flow to assess the real-time QoS provided by the network and, if necessary, generate alerts to the healthcare information system. Finally, the QoS Daemon is in charge of all the control and signalling communications between the nodes within the BWSN.

### Working principle

The suggested QoS-based network management consist of two modules, namely the QoS monitoring module and the admission control module. The QoS monitoring module embraces the following objectives: to monitor the real-time QoS supported by the network, and to detect and classify QoS degradation events. On its turn, the admission control module is in charge of managing the admission location of new sensor nodes to the network to prevent its performance degradation due to network congestion.

The QoS monitoring module analyses the incoming traffic at the sink to extract relevant metrics (e.g., the packet reception rate) in order to assess the QoS provided by the network. Then, such metrics are evaluated using time-domain techniques to detect QoS degradation events. Regarding the QoS degradation events, they can be classified into two categories: hard degradation events and soft degradation events. Hard degradation events are related to the failure of static thresholds previously defined for each metric; in such cases, the normal operation of the applications using the network may be affected. On the other hand, soft degradation events are related to variations in the metrics within the imposed thresholds. Although soft degradation events may not cause the failure of the applications using the network, they must be detected and classified. For instance, a small QoS degradation may lead to a hard degradation event if persisting the necessary time. To prevent such situations, the time-domain analysis is used not only to compute the actual value of each metric but also to investigate the metrics dynamic.

**Figure 3:**
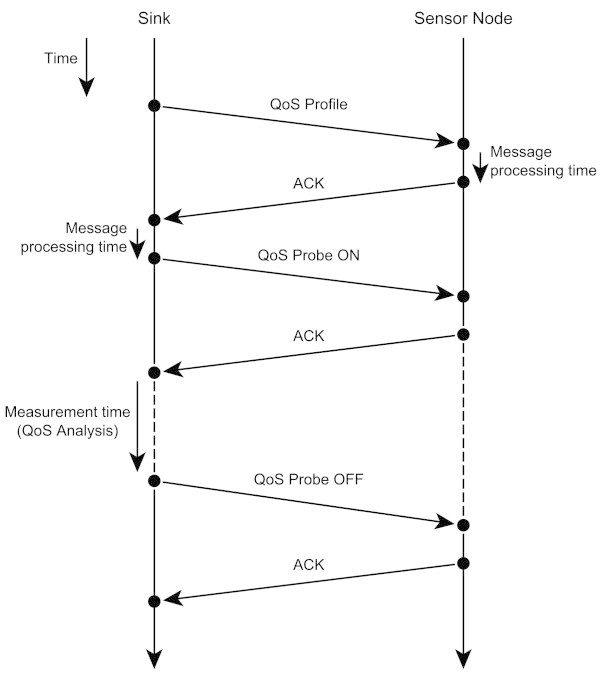
**The probing procedure performed to verify if a new node can be admitted by the network.**

Traditional admission control systems use a signalling protocol to establish reservations at all routers along the data path. This approach has to preserve per-flow state and to process per-flow reservation messages at all routers, resulting in limited scalability and high computational complexity. To avoid these problems, the proposed admission control system follows the endpoint admission control approach in the sense that the admission test is made at the edge nodes, and it is made for the entire path from the source to the sink node ([Más and Karlsson 2007]; [2008]). In this way, the proposed admission control system avoids the complexity of per-hop schemes without adding any complexity to the network nodes. The admission control is done through a “virtual sensor node” on the network, to mimic the presence of the new real node on its vicinity, and measuring how it affects the QoS provided by the network. The admission procedure, which is controlled by the healthcare providers (or who is referred to as operator”), have the following phases: first the operator phases: first the operator selects the type of the sensor node to be added to the network and its preferential location in the network; then the operator requests the system to evaluate the network in order to assess its QoS; finally, based on the information retrieved by the system, the operator decides where the node can be admitted to the network. During the network evaluation phase, the system configures the QoS Probe running on one of the sensor nodes placed in the location chosen to the new sensor node to mimic the presence of the new sensor node. Then, the QoS Probe starts sending a data flow, identical to that which will be generated by the real node, to the sink. Finally, the system analyses the changes in the network’s QoS introduced by the new data flow and reports it to the operator that decides where the new sensor node can be admitted to the network. Figure 3 shows the probing procedure and all the messages exchanged to assess if a new node can be admitted to the network.

## Time domain analysis of the QoS metrics

The QoS metrics can be considered as static or dynamic. Static metrics do not depend on the network operation and are constant along the time. On the other hand, dynamic metrics vary according to network operation and are essential to know the real network status and performance ([Xiao et al. 2004]). Regarding the dynamic metrics, they can be assessed considering only its current value, or taking into consideration its past behaviour. By taking into consideration its behaviour in the near past, it is possible to assess the metrics in view of its tendency to the near future.

In the following analysis, the dynamic metrics used to assess the QoS provided by the network are seen as time domain functions. Generically, such metrics can be modelled as: 1

where *m*(*t*) represents the measured value of the metric, *m*^∗^(*t*) represents the real value of the metric, and the noise function *wgn*(*t*) represents the metric’s variation due to random interferences and the natural fluctuations of the network.

### Computing the metrics value

After its setup time, a network tends to be stable and the metrics used to compute the network’s QoS could be found stable, despite the small fluctuations that can be observed. Thus, a moving average filter can be used to estimate the value of the metrics used to quantify the QoS provided by the network. Therefore, consider the following moving average filter: 2

where *g*(·) represents the signal to be filtered, *N* is the number of observation used in the moving average, *n* is the index of the most recent observation of the sample to be processed, and *k*=*n* if *n*<*N* or *k*=*N* in other cases. The use of moving average filters reduces the effects of random instabilities on the metric. Thus, the estimated value of the metric is represented by , and its value is computed as: 3

The number of observations used to estimate the value of each metric depends both, on the network operation and on the deployment environment. As a matter of example, consider a network deployed inside a hostile environment in which the quality of the wireless links can often vary considerably. In such case it is reasonable to use a large number of observations to estimate the metrics’s value, in other words, it is necessary to analyses the network during the last *T* seconds, being *T* the time necessary to compute the metrics’s observations being considered. Therefore, the value of *N* must be determined taking in consideration the network typical operation and the target deployment environment.

### Detecting relevant variations in the metrics

The network operation can be affected both by external events (e.g., radio interferences) and by internal events (e.g., node dead due to energy depletion). In both circumstances, these events may reflect itself as perturbations in the metrics being used to assess the network’s QoS. Detecting such perturbations is of major importance to prevent the degradation of the network’s performance.

In such context, we propose the use of two features to detect significant perturbations in the metric’s value, namely the energy of the metric differentiation 4

and an energy threshold dynamically computed as: 5

The perturbations potentially harmful to the network’s performance are detected by comparing the actual  against the *E*_*threshold*_. Thus, a potential harmful perturbation is detected if the actual energy of the metric differentiation is greater than the energy threshold, . In order to quantify the potential degradation caused by harmful perturbations, a Metric Degradation Index was defined as: 6

The *MDI* gives quantitative information about the change on the metric energy.

After calibration, the *MDI* can be used to detect potential degradation events, the higher the *MDI*, the greater is the potential degradation of the network performance. To calibrate the *MDI*, several approaches can be used, depending on the QoS monitoring strategy. One possibility consists of using the maximum *MDI* achieved during the normal network operation as a threshold to detect degradation events and fire alert messages. Other possibility is using the *MDI*’s mean value achieved during the network normal operation.

### Detecting small variations in the metrics

Although useful to detect significant perturbations on the metrics being analysed, the previous analysis is insensitive to small and monotonic variations on the metrics. In other words, the previous analysis is insensitive to variations resulting in . To detect such small variations three figures-of-merit are used, namely, the Metric Tendency (*MT*), the Zero Crossing Rate (*ZCR*) and the classification of the most recent observation of the metric as a single global minimum (*MIN*) or a single global maximum (*MAX*) within the sample being processed.

Consider the functions *h*_*i*_,*i*=1,...,*p*. The product function of these functions *h*_*i*_ is represented by . In the case of *h*_1_≡*h*_2_≡...≡*h*_*p*_≡*h*, we obtain . By *id* we denote the identity function.

The *MT* can be determined looking to the slope (*S*) of a linear regression curve obtained using the method of the least squares, as: 7

Within the sample being processed, if  the  is to decrease (*↓*), if  the  is to increase (*↑*) and, finally, if  the  is to be constant (→).

On its turn, the *ZCR* feature is defined as: 8

where *sgn* is the sign function.

Finally, to verify if the observation  is a single global minimum or a single global maximum, within the sample being processed, the following expressions are used, respectively: 9

and 10

The order of the filter (i.e., the length of the sample being processed) used to compute the previous figures-of-merit depend on the QoS monitoring policy in use. Considering a small value of *N*, the QoS monitoring system becomes very sensitive and reactive. In opposition, a QoS monitoring system less reactive must use a higher *N* value. Nevertheless, it is necessary to find equilibrium between the order of the filter and the desired reactiveness of the QoS monitoring system.

By using these three figures-of-merit it is possible to detect small and monotonic variations, over the sample being processed, on the metric being analysed. By detecting such situations, corrective measures can be taken to prevent the further degradation of the QoS provided by the network.

### QoS assessment and network performance classification

Considering the classification presented in ([Truong et al. 2006]), the QoS metrics used to assess the network’s performance can be sorted into two sets: those that need to be maximised, denoted as *m*_*max*_ and, the remaining that need to be minimised *m*_*min*_. Based on this classification, and using the features previously presented, it is possible to analyse the performance of the network and classify suspicious events.

The network performance assessment and classification if the QoS metric belongs to *m*_*max*_, is analysed using the criteria presented in the Table 1, resulting in the following rule to detect Performance Degradation (*PD*): 11

**Table 1:** **Network performance assessment and classification if QoS metric belongs to** ***m***_***max***_

					Network performance
<0	≤*E*_*threshold*_	*↓*	>0	1	Degrading
<0	≤*E*_*threshold*_	*↓*	>0	0	Not degrading or recovering
<0	≤*E*_*threshold*_	*↓*	=0	n.a.	Degrading
<0	≤*E*_*threshold*_	→ or *↑*	n.a.	n.a.	Not degrading
<0	>*E*_*threshold*_	n.a.	n.a.	n.a.	Degrading
≥0	n.a.	n.a.	n.a.	n.a.	Not degrading

On the other hand, if the QoS metric belongs to *m*_*min*_, the rule to detect *PD*, derived from Table 2, is: 12

**Table 2:** **Network performance assessment and classification if QoS metric belongs to** ***m***_***min***_

					Network performance
>0	≤*E*_*threshold*_	*↑*	>0	1	Degrading
>0	≤*E*_*threshold*_	*↑*	>0	0	Not degrading or recovering
>0	≤*E*_*threshold*_	*↑*	=0	n.a.	Degrading
>0	≤*E*_*threshold*_	→ or *↓*	n.a.	n.a.	Not degrading
>0	>*E*_*threshold*_	n.a.	n.a.	n.a.	Degrading
≤0	n.a.	n.a.	n.a.	n.a.	Not degrading

## Experiments and results

Network simulators are widely used to assess and compare the performance of WSNs ([Liang 2009]). Their use has advantages and disadvantages. They allow easy and fast network deployments, controllable and flexible scenarios and the repeatability of the obtained results. However, due to the oversimplified channel models and protocols, simulations may not represent the reality. On the contrary, real deployments avoid problems relating to simplifications of models and protocols, but such deployments are considerably harder to implement and deploy. For these reasons, a hybrid approach based on the cross level emulation and simulation tool known as COOJA ([Osterlind et al. 2006]) has been used in this work. COOJA is a flexible WSNs simulator designed for simulating networks running Contiki OS ([Dunkels et al. 2004]). The BWSN used in this work was developed with the Contiki OS and simulated on COOJA using the framework presented in ([Abreu et al. 2012]).

### Simulation setup

**Figure 4:**
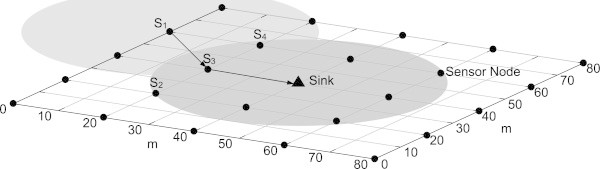
**Network deployment.** The sensor nodes are regularly distributed over an 80 m x 80 m area. Each node has a radio range of 30 m. The sink is at position (40, 40).

To assess the proposed QoS-based network management system, a case study in which a BWSN is used to monitor 25 inpatients was tested. In such context, to maximise the covered area and, at the same time, minimise the probability of collisions and the effect of funnelling to the sink, the BWSN was regularly deployed in a square area ([Younis and Akkaya 2008]), as pictured in the Figure 4. The 26 nodes (1 sink and 25 sensor nodes, one for each patient) have a radio range of 30 m. After the network setup time, which is about 60 s, each sensor node starts sending data packets at a specific rate. See Table 3 for a detailed description of both the network and the simulation configurations.

**Table 3:** **Network configuration**

Network:	
Deployment Area	80 m × 80 m
Deployment Type	5 × 5 grid (see Figure 4)
Number of Nodes	1 sink and 25 sensor nodes
Sink Position	(40,40) m
Radio Range	30 m
Radio Model	Unit Disk Graph Medium Distance Loss
Setup Time	60 s
Network Layer	IPv6 with 6LowPAN
Transport Layer	User Datagram Protocol (UDP) ([Postel 1980])
Routing Protocol	RPL with the MRHOF ([Gnawali and Levis 2012])
Logical Topology	Random
PRR Required	98%,91%
**Application:**	
Task Type	Time driven
Data Length	< 70 bytes (one packet)
Reporting Interval (s)	1, 2
**Simulation:**	
Time	1000 s

### Evaluation and results

To evaluate the proposed QoS-based network management system, this section provides several simulation and the corresponding results. The evaluation consists of two parts. First, the QoS monitoring module is used to detect and classify potential harmful events relating to QoS degradation. Then, the admission control module is used to verify if a new sensor node can be admitted by the BWSN in the requested area. In both scenarios the PRR is taken as a figure-of-merit to evaluate the QoS provided by the network. The PRR was defined as: , where *M* is the number of sensor nodes on the network, *R*_*i*_ is the number of data packets received by the sink from the node *i*, and *S*_*i*_ is the number of data packets sent by the node *i*.

#### Evaluating the QoS monitoring module

Regarding the QoS monitoring module, it was tested in order to evaluate its capacity to detect not only small and monotonic variations in the PRR (i.e., the metric being used to assess the QoS provided by the network) able to cause QoS degradation in the long term, but also sudden variations in the PRR potentially dangerous for QoS.

**Figure 5:**
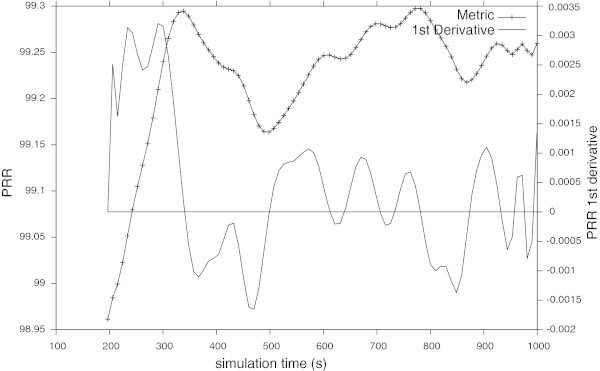
**PRR of the network in its normal operation.** The metric dynamics is represented with its first derivative.

**Figure 6:**
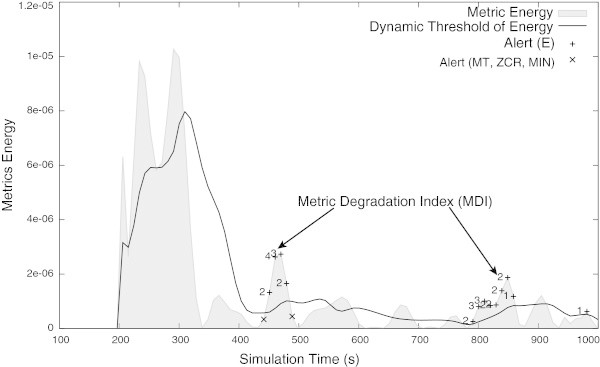
**Metric’s energy and its dynamic threshold.** It is possible to see the QoS degradation alerts and it’s *MDI*.

Figure 5 shows the PRR and its first derivative when testing the network on its normal operation. Comparing the Figure 5 with the Figure 6 it is possible to verify that, by using the features and rules of the Table 1, the QoS monitoring module is able to detect and classify different suspicious events concerning the QoSdegradation.

A detailed observation of Figure 6 permits to verify (around 440 s and 490 s) that using both, information about the metric’s dynamic and the previous defined figures-of-merit, the QoS monitoring module was able to detect slight and monotonic variations in the PRR. Concerning the detection and classification of potentially harmful QoS degradation events using the energy feature, it is important to notice that small values of the *MDI* correspond to small variations on the metric being analysed.

To verify how the QoS monitoring module responds to external interferences, such as radio interferences or link degradation, were introduced variations on the Unit Disk Graph Medium Distance Loss (UDGM-DL) radio model used within the COOJA simulator. The UDGM-DL is a wireless channel model where the transmission range is modelled as an ideal disk. The sensor nodes outside this disk don’t receive packets. The sensor nodes inside the disk receive packets accordingly a probability that depends from several parameters according the following equation: , where *Tx* represents the success ratio of the transmission, the *Rx* represents the success ratio of the reception, *d* is the distance between the two sensor nodes and *d*_*max*_ is the maximum transmission range. The external interferences were simulated by changing the *Rx* and *Tx* success ratios of the UDGM-DL radio model as shown in Table 4.

**Table 4:** **UDGM-DL radio model used to simulate radio interferences, see Figure** 7

Simulation time on the Figure 7 (s)	0	400	500	600	700	800	900
Tx and Rx success ratio (%)	100	75	100	50	75	80	100

In order to introduce selectivity on the detection of significant degradation events, and at the same time avoid alarm fatigue due to over-alarming, a threshold was introduced in the *MDI*. In this way, the maximum *MDI* value obtained during the normal network operation (i.e., *MDI*=4) was used as a minimum limit to detect a degradation event and fire the corresponding alarm. In other words, degradation events are detected and fired if the *MDI* value exceeds the one used to calibrate the QoS monitoring system. In addition, a real application of the proposed method can introduce other mechanisms to improve selectivity.

**Figure 7:**
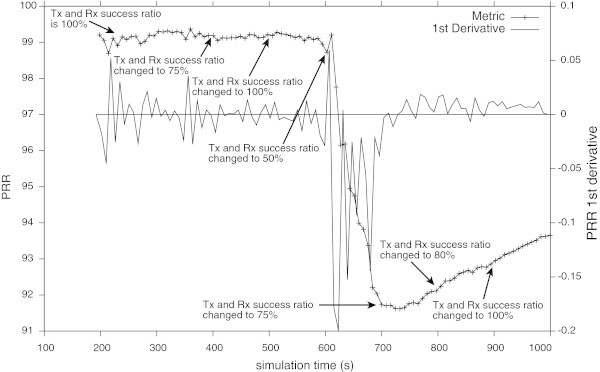
**PRR of the network exposed to a hostile environment in terms of the radio channel with interferences as indicated in Table** 4 . The metric dynamics is represented with its first derivative.

**Figure 8:**
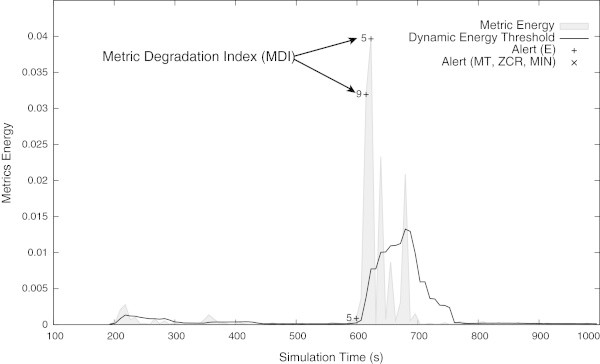
**Metric’s energy and its dynamic threshold.** It is possible to see the QoS degradation alerts and it’s *MDI*. Comparing with the Figure 6, it is possible to confirm that higher *MDI* greater is the degradation of the network performance.

The Figure 7 shows that the radio interferences introduced in the simulation have produced a strong degradation in the PRR and consequently in the QoS provided by the network. Analysing the Figure 7, and comparing it with the Figure 8, it is possible to confirm that the QoS monitoring module has detected the QoS degradation at its very beginning. More, from these results it is possible to confirm that high values of the *MDI* suggest that the metric being analysed will suffer a strong degradation. Comparing the Figure 6 (i.e., the network normal operation) with the Figure 8 (i.e., the network suffering a strong perturbation), it is possible to confirm that the higher *MDI* the greater is the degradation of the network performance. By detecting the QoS degradation events at its beginning, the QoS monitoring module can be also used to fire messages informing the network manager about the possible QoS degradation. On its turn, the network manager can take measures to mitigate the effects of the QoS degradation even before it takes critical values.

In addition to the present analysis, the proposed method can be used to assess the performance of each individual node within the network. This possibility, while requiring additional computational power and memory, allows obtaining additional information about the network performance. In particular, monitoring individual nodes is crucial to identify the source of the performance degradation events, which is impossible when analysing the overall performance of the network.

#### Evaluating the admission control module

To test the admission control module, the aforementioned probing procedure was used to evaluate if a new sensor node can be admitted to the network in the place under test. The probing procedure starts on request (e.g., by a healthcare professional) and stops when one of the following conditions is observed: the metric value crosses a pre-established limit or the metric tendency (to increase or decrease) is interrupted, in other words  for the metrics belonging to *m*_*max*_, and  for the metrics belonging to *m*_*min*_.

Regarding the evaluation of the admission control module, two scenarios were considered. For the first, each sensor node within the network was set to generate constant bit rate traffic of about 0.5 packets per second, where the network is considered uncongested and able to admit a new sensor node while maintaining a PRR of, at least, 98%. In the second scenario, the sensor nodes were set to send 1 data packet per second to the network, and it has to fulfil a PRR of, at least, 91%. Herein, the network is congested and unable to admit a new sensor node while maintaining the requested QoS.

**Figure 9:**
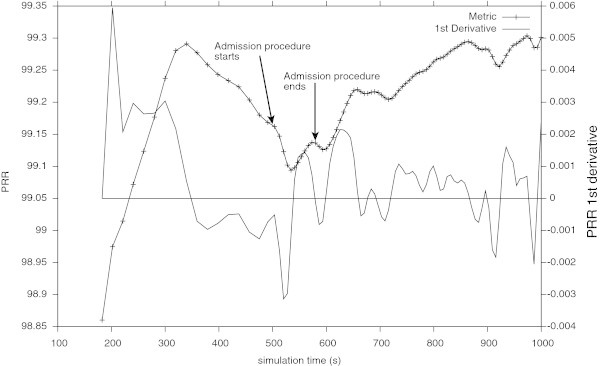
**PRR of the uncongested network during the admission control procedure (QoS Probe ON) and its derivative.** The probe procedure ends when  at 590 s.

**Figure 10:**
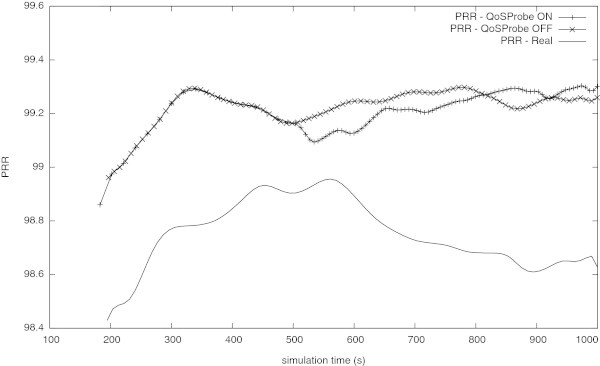
**PRR of the uncongested network in its normal operation (QoS Probe OFF), with the QoS Probe ON and with the new node (Real).**

For the uncongested network, analysing the Figure 9 allows to withdraw the results summarised in the Table 5. The admission control procedure starts at simulation time of about 500 s and lasts until the  which happens at simulation time of about 590 s, as shown in the Figure 9. Now, the admission control module computes the PRR that is taken as an estimation of the PRR if the real sensor node was added to the network. Since the QoS Probe do not introduce the same effects (e.g., radio interference) on the network as a real sensor node, this estimation is considered as the upper limit of the real PRR. The PRR estimated to be off about 99.14%, i.e., greater than the pre-established limit of 98%, and the decision about the admission of the new node is positive. After the admission control procedure, the new sensor node was added to the network. Then the network was evaluated and the PRR depicted in the Figure 10 was obtained. Such results, confirm the estimation obtained with the admission control procedure, showing that the new sensor node can be added to the network, without degradation the PRR bellow the pre-established limit of about 98%.

**Table 5:** **Results obtained from the admission control procedure after a measurement time of about 90 s, see Figure** 9

Beginning of	End of measurement	PRR at the beginning	PRR at the end	Measurement time
the measurement		of the measurement	of the measurement	
500 (s)	590 (s)	99.16%	99.14%	90 (s)

It’s possible to conclude that the PRR of the uncongested network with the new node will be, on the best, of about 99.14%.

**Figure 11:**
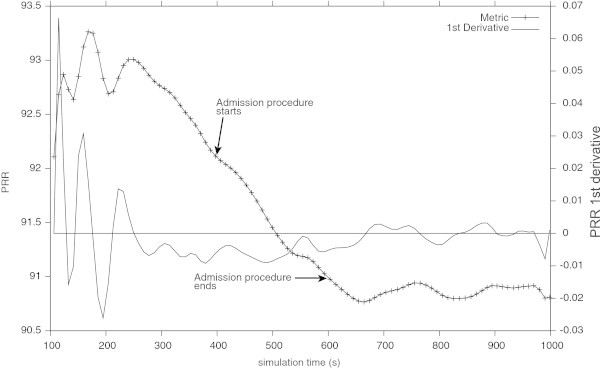
**The PRR of the congested network during the admission control procedure (QoS Probe ON) and its first derivative.** The probe procedure ends when the *P* *R* *R*≤91*%* at 600 s.

Regarding the evaluation of the admission procedure in the scenario of a congested network, the results achieved are summarised in the Table 6. The admission control procedure starts at simulation time of about 400 s and lasts until the *PRR*≤91*%*, witch happens at simulation time of about 600 s, as shown in the Figure 11. Here, the proposed admission control procedure was able to predict that the congested network was unable to accommodate the new node. In that case the PRR estimated drops below the pre-established limit of 91% and the decision to admit the new node is negative.

**Table 6:** **Results obtained from the admission control procedure, after a measurement time of about 200 s, see Figure** 11

Beginning of	End of measurement	PRR at the beginning	PRR at the end	Measurement time
the measurement		of the measurement	of the measurement	
400 (s)	600 (s)	92.07%	91%	200 (s)

It is possible to conclude that the PRR of the congested network with the new node will be less than the pre-established limit of about 91%.

**Figure 12:**
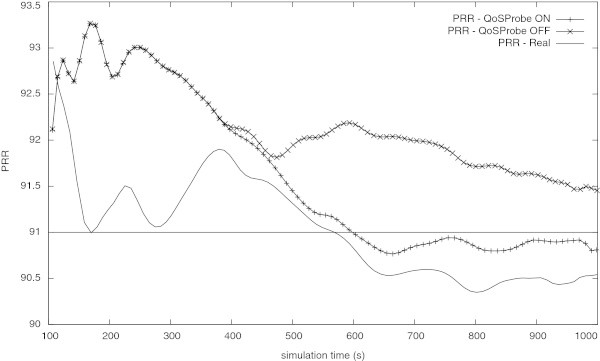
**PRR of the congested network in its normal operation (QoS Probe OFF), with the QoS Probe ON and with the new node (Real).**

In similarity to what was done in the previous experiment, the new sensor node was added to the network. Then, the network was evaluated to validate the values previously estimated and, consequently, to confirm the decision that was made. The Figure 12 shows the results obtained. Such results confirm the estimation presented in the Table 6 and confirms that the new sensor node cannot be added to the network without degrading the PRR bellow the pre-established limit of about 91%.

## Conclusion

Biomedical wireless sensor networks have to fulfil high levels of confidence and reliability in order to be used in patient monitoring scenarios, particularly those where data collection is vital for diagnosis or to improve the quality of care provided in hospital units and nursing houses. This paper presented a QoS-based network management system that comprises two modules namely, the QoS monitoring module and the admission control module. The QoS monitoring module is able to detect and classify suspicious events regarding the degradation of the QoS provided by the network. More, by using not only the metric value but also its dynamic, the QoS monitoring module is able to detect and classify QoS degradation events even before the metric reaches critical values. By using this ability, the QoS monitoring system can be used to fire advertising messages to the network manager informing about the potential QoS degradation event. On its turn, the network manager can take measures to mitigate the effects of such events, preventing the performance of the network from degrading. Regarding the admission control system, it can be used to assess if the network can admit a new sensor node, i.e., a new patient being monitored. By using a “virtual sensor node to mimic the presence of the new real sensor node on its neighbourhood while assessing the network, the proposed admission control system is able to test the network even from a remote location. In this way, the proposed QoS-based network management system allows to monitor and manage the network from a centralised location, which is an important feature regarding patient monitoring scenarios in hospital facilities or nursing homes.
